# CRKL but not CRKII contributes to hemin‐induced erythroid differentiation of CML

**DOI:** 10.1111/jcmm.18308

**Published:** 2024-04-29

**Authors:** Chunmei Guo, Xinxin Lv, Qiuling Zhang, Lina Yi, Yingying Ren, Zhaopeng Li, Jinsong Yan, Shanliang Zheng, Ming‐Zhong Sun, Shuqing Liu

**Affiliations:** ^1^ Department of Biotechnology & Liaoning Key Laboratory of Cancer Stem Cell Research, College of Basic Medical Sciences Dalian Medical University Dalian Liaoning China; ^2^ Department of Biochemistry, College of Basic Medical Sciences Dalian Medical University Dalian Liaoning China; ^3^ Department of Hematology, The Second Affiliated Hospital of Dalian Medical University Institute of Stem Cell Transplantation of Dalian Medical University Dalian Liaoning China

**Keywords:** CML, erythroid differentiation, miR‐429‐CRKL axis, Raf/MEK/ERK pathway

## Abstract

Destruction of erythropoiesis process leads to various diseases, including thrombocytopenia, anaemia, and leukaemia. miR‐429‐CT10 regulation of kinase‐like (CRKL) axis involved in development, progression and metastasis of cancers. However, the exact role of miR‐429‐CRKL axis in leukaemic cell differentiation are still unknown. The current work aimed to uncover the effect of miR‐429‐CRKL axis on erythropoiesis. In the present study, CRKL upregulation was negatively correlated with miR‐429 downregulation in both chronic myeloid leukaemia (CML) patient and CR patient samples. Moreover, CRKL expression level was significantly decreased while miR‐429 expression level was increased during the erythroid differentiation of K562 cells following hemin treatment. Functional investigations revealed that overexpression and knockdown of CRKL was remarkably effective in suppressing and promoting hemin‐induced erythroid differentiation of K562 cells, whereas, miR‐429 exhibited opposite effects to CRKL. Mechanistically, miR‐429 regulates erythroid differentiation of K562 cells by downregulating CRKL via selectively targeting *CRKL*‐3′‐untranslated region (UTR) through Raf/MEK/ERK pathway. Conversely, CRKII had no effect on erythroid differentiation of K562 cells. Taken together, our data demonstrated that CRKL (but not CRKII) and miR‐429 contribute to development, progression and erythropoiesis of CML, miR‐429‐CRKL axis regulates erythropoiesis of K562 cells via Raf/MEK/ERK pathway, providing novel insights into effective diagnosis and therapy for CML patients.

## 1 INTRODUCTION

Haematopoiesis is a precisely modulated multistep process including haematopoietic stem cells (HSC) self‐renewal and haematopoietic stem/progenitor cell differentiation.^1^, ^2^ Haematopoiesis occurs in the bone marrow (BM), where HSC with self‐regenerative and highly proliferative capacity give rise to multipotent progenitors (MPPs) that can differentiate into the common myeloid progenitors (CMP) or common lymphoid progenitors (CLP).^3^ The CMP differentiation process is called myelopoiesis which can produce erythrocytes, megakaryocytes, granulocytes and macrophages.^4^ Erythropoiesis is important parts of haematopoiesis.^5^, ^6^ which is a complex and dynamic process whereby mature red blood cells (RBCs) are produced from HSC and progenitor cells. Normal erythropoiesis produces about 10^11^ new RBCs every day in an adult human through the commitment of HSC into erythroid progenitors, which subsequently differentiate into mature RBCs.^7^, ^8^ Erythropoiesis plays an important role in the maintenance of RBCs mass and the delivery of oxygen to tissues. This process occurs almost exclusively in the BM microenvironment of adult mammals where both systemic and local cues regulate erythropoiesis.^7^ Destruction of erythropoiesis processes leads to various diseases, including thrombocytopenia, anaemia and leukaemia, including chronic myeloid leukaemia (CML). Understanding the regulatory mechanism of erythropoiesis can lead to characterizing novel modulators and developing new methods for treatment of blood‐related diseases.

CML is a clonal myeloproliferative pluripotent haematopoietic stem cell malignancy disorder characterized by the expression of B‐cell receptor/v‐abl Abelson murine leukaemia viral oncogene (*BCR/ABL1*) fusion gene,^9^, ^10^ which is generated from the Philadelphia chromosome translocation of chromosome 9–22.^11^, ^12^ BCR‐ABL is the molecular hallmark of CML with tyrosine kinase activity that can potentially activate multiple signal transduction pathways, resulting in abnormal cell proliferation, apoptosis, migration, invasion and differentiation.^13^, ^14^, ^15^ The delay of differentiation and maturation is considered to be a characteristic of leukaemia, inducing leukaemia cell differentiation and breaking through the barrier of differentiation and maturation has become a research hotspot in basic medical research and its clinical translation.^16^ The K562 is human leukaemia cell derived from the pleural effusion of a CML woman patient in terminal blast crisis.^17^, ^18^ K562 cells behave more like undifferentiated early pluripotent haematopoietic progenitors, and have been widely used as a model for studying haematological cell differentiation due to its ability to express specific markers of granulocytic, monocytic, erythroid and megakaryocytic lineages.^19^, ^20^

The CT10 regulation of kinase (CRK) adapter protein family is involved in intracellular signal transduction. The CRK family members were the first identified adaptor proteins, which connect with upstream molecules through their C‐terminal SH2 domain and with downstream molecules through their N‐terminal SH3 domain.^21^ CRK consists of cellular homologues CRKI, CRKII and CRK‐like (CRKL) which are ubiquitously expressed and conserved across eukaryotic organisms.^22^ CRKI and CRKII were originally described as splice variants, while CRKL is encoded by another homologous gene. CRKI is composed of one SH2 domain and one SH3 domain, CRKII and CRKL are composed of one SH2 domain, one SH3N and one SH3C domains.^22^, ^23^, ^24^ CRKII and CRKL are highly similar in sequence and both possess tyrosine phosphorylation sites that can be phosphorylated by BCR‐ABL to activate signalling pathways,^22^, ^23^ suggesting CRKII and CRKL have overlapping functions. CRKII and CRKL contains a variety of linkages for docking BCR‐ABL, p130Cas, Dock180, GAB, ABL‐1, Pax, GEF, C3G and SOS to form localized complexes critical for cell proliferation, survival, adhesion and metastasis.^25^, ^26^, ^27^ CRKII and CRKL deregulation have been proved to be involved in the development and progression of a variety of cancers.^28^, ^29^, ^30^ Nevertheless, a few studies report the association of CRK with differentiation: CRK could induce pheochromocytoma PC12 cell differentiation^31^, ^32^; CRKII and CRKL could synergistically increase RANKL‐induced osteoclast differentiation.^33^ However, the precise roles and underlying mechanisms of CRKL and CRKII in leukaemic cell differentiation are still not reported.

MicroRNAs (miRNAs) are 18–24 nucleotide small non‐coding RNAs that negatively regulate gene expression by directly degrading mRNA or by suppressing post‐transcriptional protein translation by binding to the 3′‐untranslated region (3′‐UTR) of targeted mRNAs, which play important roles in cell proliferation, differentiation, metastasis and apoptosis.^34^, ^35^, ^36^ MiRNAs might function as tumour promoter or suppressor in tumorigenesis and tumour malignancy.^37^ miR‐429, a member of miR‐200 family, is located on chromosome 1p36.^38^ miR‐429 dysregulation is involved in the development, progression, epithelial–mesenchymal transition (EMT), metastasis and drug resistance of various cancers.^39^ It functions either as a tumour suppressor or promoter for certain cancers depending on the particular type of tumour cells/tissues.^39^ Our previous study showed that miR‐429 negatively regulated CRKL expression by binding to *CRKL*‐3′‐UTR,^40^, ^41^ miR‐429 suppressed the migration and invasion of hepatocellular carcinoma (HCC) and clear cell renal cell carcinoma (ccRCC) cells by targeting CRKL via Raf/MEK/ERK pathway^41^ and SOS1/MEK/ERK/MMP2/MMP9 pathway.^40^ However, the exact role and underlying molecular mechanism of miR‐429‐CRKL axis in erythroid differentiation of CML is unknown.

In the present study, we report a novel biological function of miR‐429‐CRKL axis in erythroid differentiation of CML. We found that CRKL was upregulated and miR‐429 was downregulated in CML samples. CRKL expression level was lower, while miR‐429 expression level was higher in CML complete remission (CR) patients than in corresponding CML patients. Meanwhile, CRKL upregulation was negatively correlated with miR‐429 deficiency in CML. miR‐429 promoted while CRKL suppressed erythroid differentiation of K562 cells. Further investigation revealed that miR‐429‐CRKL axis regulates erythroid differentiation of K562 cells via Raf/MEK/ERK pathway. Conversely, CRKII had no effect on erythroid differentiation of K562 cells. Our study has uncovered a novel miR‐429‐CRKL‐Raf/MEK/ERK regulatory pathway in erythroid differentiation of CML and partially elucidated the molecular mechanism underlying erythroid differentiation in CML cells. miR‐429‐CRKL axis may serve as a potential target for therapeutic treatment and prognosis of CML disease.

## 2 MATERIALS AND METHODS

### 2.1 Patients and blood samples

A total of 34 CML primary patient BM samples, 6 CML CR patient BM samples and 13 healthy subject normal peripheral blood (PB) samples were collected from the Department of Haematology, The Second Affiliated Hospital of Dalian Medical University, Dalian, China. The mononuclear BM and PB cells were separated from the CML patients, non‐malignant leukaemia patients and healthy subject normal samples, respectively, all mononuclear cell specimens were frozen in liquid nitrogen immediately after separation and stored at −80°C prior to RNA isolation. The patients were treated with imatinib, nilotinib, dasatinib, the treatment response was assessed by molecular monitoring through quantitative real‐time RT‐PCR (qRT‐PCR), the complete molecular response (CMR) is defined as MR4.5. All experiments involving human patient samples were conducted according to the ethical policies and the procedures approved by Medical Ethics Committee of Dalian Medical University (Approval no. 2019‐014). Meanwhile, informed consent was obtained from all patients. All experiment methods were performed in accordance with the relevant guidelines and regulations.

### 2.2 qRT‐PCR assay

Total RNA was extracted from patient samples and K562 cells using Trizol™ reagent (TransGen, ET111‐01, China) and reversely transcribed into cDNA using EasyScript® One‐Step gDNA Removal and cDNA Synthesis SuperMix (TransGen, AE311‐03, China). qRT‐PCR was then performed using TransStart Tip Green qPCR SuperMix (TransGen, TG‐AQ601‐02, China) with Step‐one Real‐time PCR System (ThermoFisher, 273021072, USA). snRNA U6 and *GAPDH*, *ACTB* were used as internal references for miR‐429 and mRNA, respectively. The relative expression levels of genes in different groups of patient samples and cells were analysed using the 2^−△△CT^ method. The specific primers of targeting genes are provided in Table S1.

### 2.3 Benzidine staining assay

To induce erythroid differentiation, K562 cells were treated with 50 μM Hemin (Sigma, BCBD3516V, USA). Benzidine staining can reflect the haemoglobin production of cell, the principle is that haemoglobin or heme in red blood cells can make H_2_O_2_ release new ecological oxygen, colourless biphenyls are oxidized to benzidine blue, which has good specificity for late differentiation or mature red blood cells. 4 × 10^5^ K562 cells in 2 mL RPMI‐1640 medium supplemented with 15% FBS were seeded into a well of 6‐well plate, then treated with 20, 40, 60, 80 and 100 μM hemin (Sigma, BCBD3516V, USA) in a humidified incubator with 5% CO_2_ at 37°C for 0, 24, 48 and 72 h before harvesting and washing once with PBS. Benzidine dihydrochloride solution was prepared with 30% acetic acid containing 0.2% H_2_O_2_, then 1 μL benzidine dihydrochloride solution was directly added to 9 μL cell suspension, incubated at room temperature (RT) for 5 min, and immediately imaged by inverted fluorescence microscope (Olympus, 3C80009, Japan) with 200× magnification. Benzidine‐positive cells were stained blue, while benzidine‐negative cells were light yellow.

### 2.4 Establishment of CRKL knockdown monoclonal K562 cells

For CRKL knockdown, targeting shRNAs were designed according to *CRKL* sequence (Genbank: NM_005207.3) using Invitrogen, siDirect and Whitehead software. Out of three *CRKL*‐specific shRNA double‐stranded oligodeoxyribonucleotides, the optimal shRNA for CRKL knockdown was with the sense and antisense sequences of 5′‐GTCACAAGGATGAATATAA‐3′ and 5′‐TTATATTCA TCCTTGTGAC‐3′. One non‐targeting shRNA with the sense and antisense sequences of 5′‐GTTCTCCGAACGTGTCACGT‐3′ and 5′‐ACGTGACACGTTCGGAGAAC‐3′ was used for negative control (NC). The constructed corresponding vectors were named as pGPU6/GFP/Neo‐shRNA‐CRKL and pGPU6/GFP/Neo‐shRNA‐NC, then were transfected into K562 cells using Lipofectamine™ 2000 (Invitrogen, 2028090, USA) according to the manufacturer's instructions. The K562 cells stably transfected with pGPU6/GFP/Neo‐shRNA‐CRKL or pGPU6/GFP/Neo‐shRNA‐NC were screened against 400 μg/mL G418 (ThermoFisher, 11811023, USA) for 14 days, respectively. Monoclonal K562‐shRNA‐NC and K562‐shRNA‐CRKL cells were obtained by limited dilution screening against G418 selection. Then, monoclonal K562‐shRNA‐NC and K562‐shRNA‐CRKL cells were grown in RPMI‐1640 medium supplemented with 15% FBS and 250 μg/mL G418 in a humidified incubator at 37°C with 5% CO_2_.

### 2.5 Plasmid construction, siRNA design and transient transfection

To overexpress CRKL in K562 cells, the full‐length coding sequence of *CRKL* was first amplificated by RT‐PCR using the forward primer 5′‐TATCTAGAGCCACCATGTCCTCCGCCAGGTT‐3′ and reverse primer 5′‐CGTTCGAAGGGCCTCGTTTTCATCTGGGTTT‐3′, then cloned into the *BstB* I and *Xba* I sites of PCDH‐EF1‐MCS‐T2A‐Puro vector. The recombinant PCDH‐EF1‐MCS‐T2A‐Puro‐CRKL expression vector was used for overexpressing CRKL in K562 cells by transiently transfected into K562 cells. For miR‐429 overexpression and knockdown, the oligonucleotides of miR‐429 mimic, miR‐NC mimic, miR‐429 inhibitor and miR‐NC inhibitor were also transiently transfected into K562 cells.

For CRKII knockdown, targeting siRNAs (small interfering RNA) were designed based on the *CRKII* sequence (Genbank: NM_016823.3, siCRKII‐1: 5′‐CCAGAATGGGCCCATATAT‐3′, siCRKII‐2: 5′‐GCGAGTCCCCAATGCCTAC‐3′). One day before transfection, 3 × 10^5^ cells/well in 2 mL RPMI‐1640 supplemented with 15% FBS were seeded into a 6‐well plate, and the siRNA mixtures (3 μL siCRKII‐1 + 3 μL siCRKII‐2) were transiently transfected into K562 cells using 5 μL Lipofectamine™ 2000 (Invitrogen, USA) according to the manufacturer's instructions for 48 h at 37°C with 5% CO_2_.

### 2.6 Western blotting assay

Each group of cells was harvested and washed with PBS, then total protein was extracted using RIPA buffer (50 mM pH 8.0 Tris–HCl, 150 mM NaCl, 1% Triton X‐100, 0.5% sodium deoxycholate and 0.1% SDS) supplemented with 1 mM Na_3_VO_4_, 1 μg/mL leupeptin and 0.5 mM PMSF. The supernatant was collected by centrifugation at 12,000 rpm for 15 min at 4°C. Equal amount of each protein sample to be determined by Bradford assay were boiled for 5 min in loading buffer and separated by 10% SDS‐PAGE. The protein bands were transferred onto a nitrocellulose membrane, blocked with 5% (w/v) skim milk in TBST buffer (pH 7.5; 100 mM NaCl, 50 mM Tris‐HCl and 0.1% Tween‐20) for 3 h at RT and incubated with primary antibodies at 4°C overnight. Then, the nitrocellulose membrane was washed with TBST for 3 × 10 min, incubated with the secondary antibody conjugated for 3 h at RT and washed again with TBST for 3 × 10 min. Protein bands were visualized by ECL (Abbkine, BMU101‐CN, China) and analysed using Bio‐Rad ChemiDoc™ MP system (Bio‐Rad, 731BR00277, USA). ACTB and GAPDH were used as internal reference.

### 2.7 Isobaric tags for relative and absolute quantitation (iTRAQ) proteomic assay

3 × 10^7^ K562‐shRNA‐NC and K562‐shRNA‐CRKL group cells were harvested in three independent experiments, and centrifugated at 1000 rpm for 5 min and then the cell pellets were washed with ice‐cold PBS. Total protein was extracted from each group of cells using SDT buffer. The SDT buffer was added to the sample and boiled for 15 min, then the supernatant was collected by centrifugation at 12,000 *g* for 15 min at 4°C and quantified by a BCA Protein Assay Kit. Protein samples (20 μg) were mixed with 5 × loading buffer, boiled for 15 min and separated by 12.5% SDS‐PAGE. Protein bands were visualized by Coomassie Blue R‐250 staining. The subsequent steps including filter‐aided sample preparation (FASP Digestion), iTRAQ labelling, peptide fractionation with strong cation exchange (SCX) chromatography and LC–MS/MS analysis were performed by the Research Center for Proteome Analysis, Shanghai Institutes for Biological Sciences according to the standard method.^42^

### 2.8 cDNA microarray assay

1 × 10^7^ K562‐shRNA‐NC and K562‐shRNA‐CRKL group cells were harvested for total RNA extraction using Trizol™ reagent (Life Sciences). The RNA concentration and quality were assessed using a NanoDrop 2000 spectrophotometer (Thermo, S863, USA) and 1.5% denaturing agarose gel electrophoresis. cDNA was synthesized using SuperScript II kit and purified by QIAGEN RNeasy Mini Kit. cRNA was created using a Genechip IVT labelling kit. The biotin‐labelled fragmented cRNA (≤200 nt) was hybridized at 45°C for 16 h on a Affymetrix Genechip (Human Transcriptome Array 2.0). All the arrays were imaged by a 3000 7G Scanner and processed by Affymetrix Genechip Operating Software. The random variance model (RVM) *t*‐test was performed to screen the differentially expressed genes between the K562‐shRNA‐NC and K562‐shRNA‐CRKL group cells.

### 2.9 Statistical analysis

Statistical analysis was performed using GraphPad Prism 6.0 software. Student's *t*‐test was performed to measure the differences between two groups. The correlation between the expression of CRKL and miR‐429 was analysed using the Spearman's rank correlation coefficient. Differences with *p* < 0.05 were considered statistically significant.

## 3 RESULTS

### 3.1 Expression patterns and the correlation of miR‐429 and CRKL in CML patients

**FIGURE 1:**
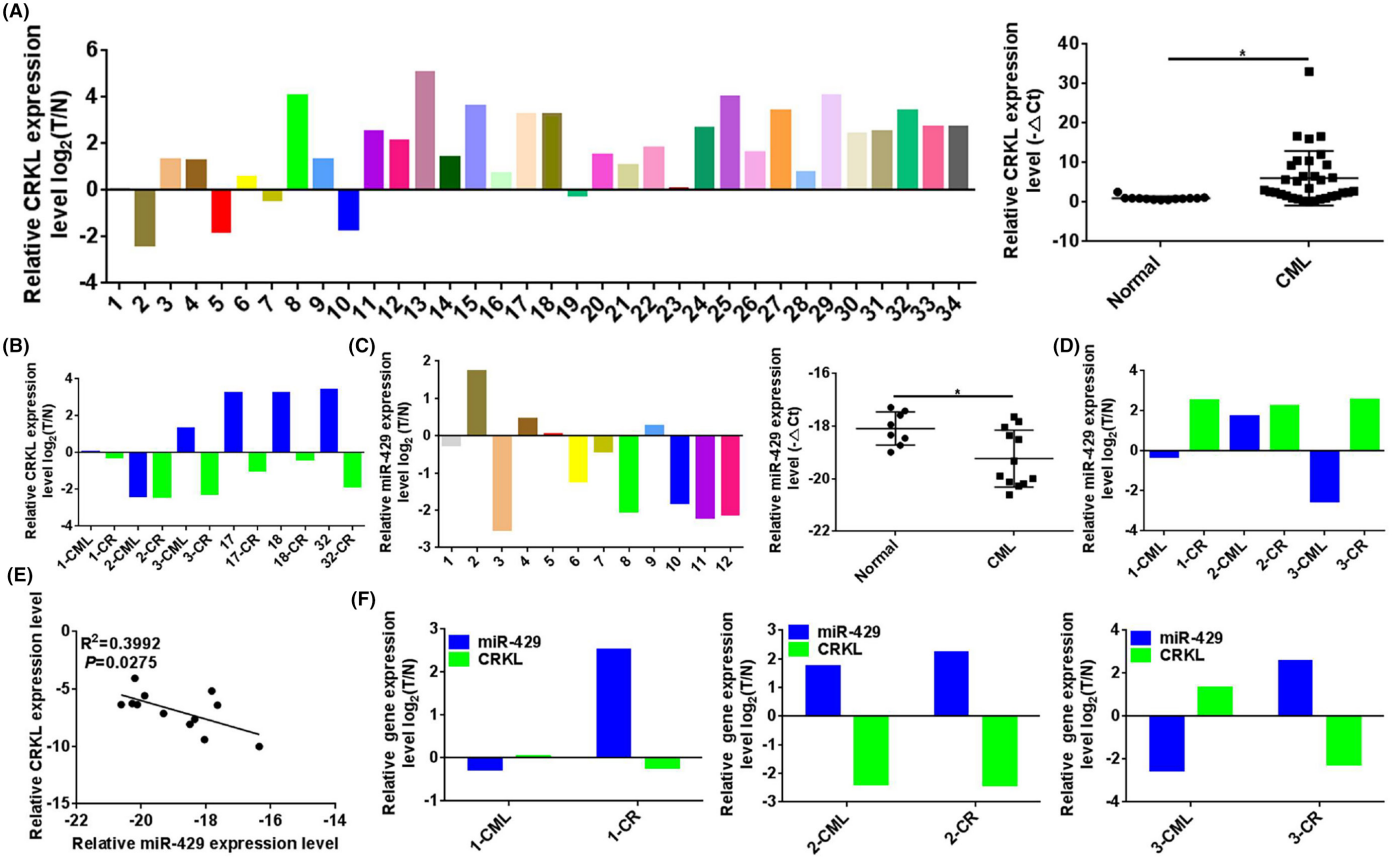
The expression patterns of CT10 regulation of kinase‐like (CRKL) and miR‐429 in chronic myeloid leukaemia (CML) patient samples. (A) CRKL was overexpressed in CML patients. (B) CRKL was downregulated in CML CR patients compared with the corresponding CML primary patients. (C) miR‐429 was poorly expressed in CML patients. (D) miR‐429 was upregulated in CML CR patients compared with the corresponding CML primary patients. (E) The CRKL expression level was negatively correlated with miR‐429 in CML patients. (F) A negative correlation was established for CRKL upregulation with miR‐429 downregulation in three pairs of CML primary and CR patient samples. *Refer to *p* values < 0.05.

To investigate the potential role of CRKL in CML, we detected the expression levels of CRKL in 34 CML primary patient BM samples, 6 CML CR patient BM samples and 13 normal PB samples by qRT‐PCR. Our results showed that CRKL was almost universally overexpressed in CML patient BM samples (29 out of 34), the mRNA expression level of CRKL was upregulated 5.1‐fold (*p =* 0.0115, Figure 1A) in CML patient BM samples compared with normal samples. We further compared the expression level of CRKL in six pairs of CML primary and CR patient samples. Interestingly, the mRNA expression level of CRKL was lower in CR patient samples than in the corresponding CML primary patient samples (Figure 1B). Our results indicated that CRKL was highly expressed in CML, the expression level of CRKL was decreased after CR of CML patients which played a crucial role in the development and progression of CML, it might be a potential diagnostic and therapeutic biomarker for CML.

Furthermore, we measured the expression levels of miR‐429 in 12 CML primary patient BM samples, 3 CML CR patient BM samples and 8 normal PB samples by qRT‐PCR. miR‐429 was significantly downregulated in CML patients (8 out of 12), the expression level of miR‐429 was downregulated 1.1‐fold (*p =* 0.0156, Figure 1C) in CML patient BM samples compared with normal samples. We further compared the expression level of miR‐429 in three pairs of CML primary and CR patient samples. Interestingly, the expression level of miR‐429 was higher in CR patient samples than in the corresponding CML primary patient samples (Figure 1D). Our results suggested that miR‐429 displayed a comparatively lower expression in CML patient samples, the expression level of miR‐429 was increased after CR of CML patients and it might be a potential biomarker for CML progression.

We further analysed the inter‐correlation of miR‐429 and CRKL expression level changes in CML patient samples. As shown in Figure 1E, the expression level of CRKL was negatively correlated with miR‐429 in CML patients (*R* ^2^ = 0.3992, *p* = 0.0275). Moreover, we further analysed the inter‐correlation of miR‐429 and CRKL expression level changes in three pairs of CML primary and CR patient samples. A negative correlation was also established for CRKL upregulation with miR‐429 downregulation in three pairs of CML primary and CR patient samples (Figure 1F). Our results demonstrated that miR‐429 expression was negatively correlated with CRKL expression, and the dysexpressions of CRKL and miR‐429 were closely correlated in affecting CML malignancy.

### 3.2 miR‐429 is upregulated while CRKL is downregulated during hemin‐induced erythroid differentiation of K562 cells

**FIGURE 2:**
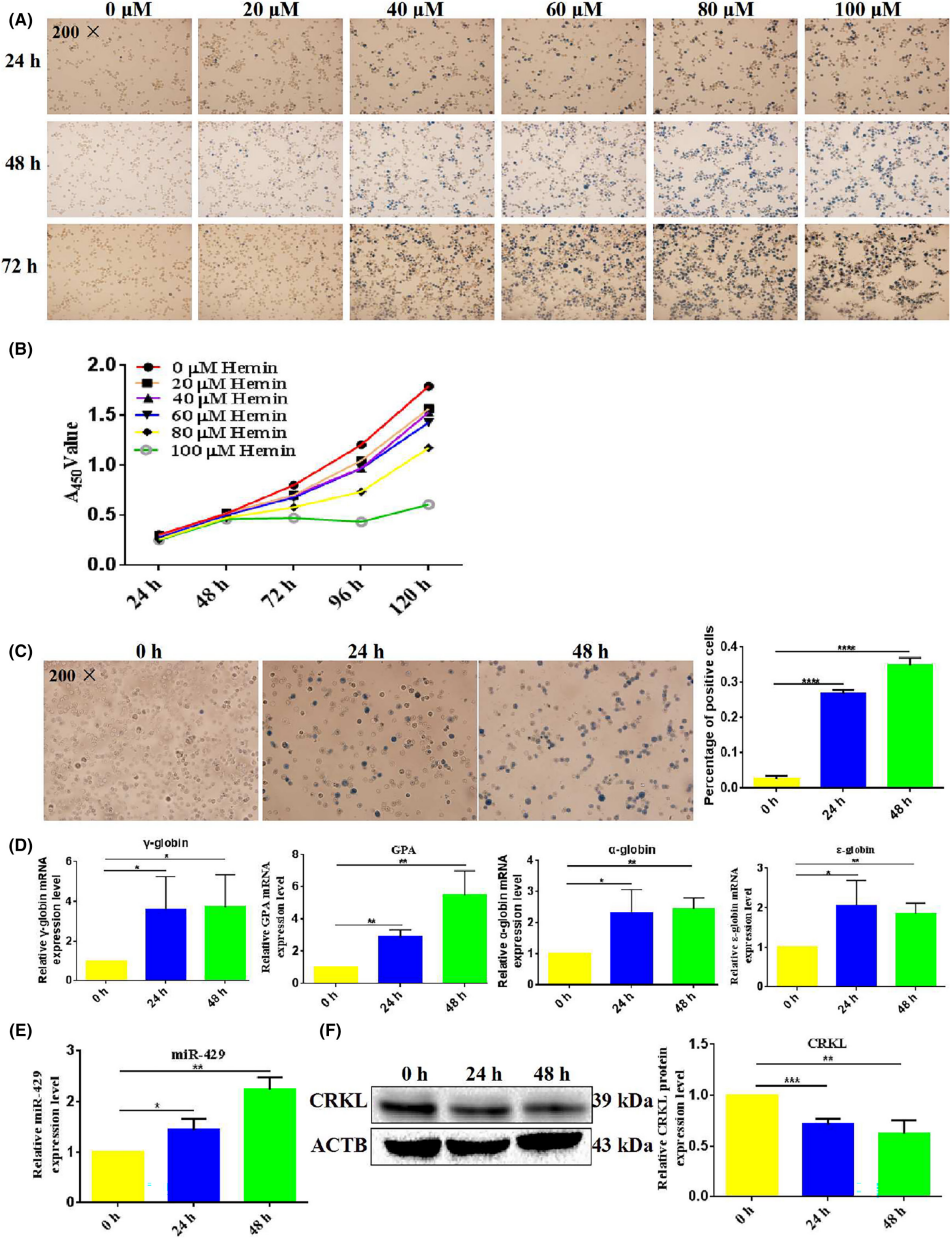
The expression patterns of CT10 regulation of kinase‐like (CRKL) and miR‐429 during hemin‐induced erythroid differentiation of K562 cells. (A) Hemin induced erythroid differentiation of K562 cells in a dose‐ and time‐dependent manner by benzidine staining assay. (B) Hemin inhibited viability and proliferation abilities of K562 cells by CCK8 assay. (C) The benzidine‐positive K562 cells induced by 40 μM hemin. (D) γ‐globin, GPA, α‐globin and ε‐globin mRNA expression levels were detected in hemin‐induced K562 cells by qRT‐PCR. (E) miR‐429 expression level was measured in hemin‐induced K562 cells by qRT‐PCR. (F) CRKL protein expression level was measured in hemin‐induced K562 cells by western blotting (WB) assay. *, **, ***, ****Refer to *p* values <0.05, 0.01, 0.001, 0.0001.

K562 cells can be differentiated into erythroid cells by treatment with hemin, we first screened the appropriate concentration of hemin which could induce erythroid differentiation of K562 cells. After treatment with hemin, K562 cells showed significant increases in the number of benzidine‐positive cells in a dose‐ and time‐dependent manner (Figure 2A). Different concentrations of hemin could induce erythroid differentiation of K562 cells, but showed different induction efficiency, so we further detected the effects of hemin at different concentrations on the viability and proliferation abilities of K562 cells using CCK8. As shown in Figure 2B, with the increase of hemin concentration, the inhibition degree of K562 cell proliferation was strengthened, meanwhile, 80 and 100 μM hemin caused K562 cell death. According to the above results, the positive rate of benzidine staining in K562 cells increased with increasing hemin concentration, while K562 cell proliferation was inhibited with increasing hemin concentration, so we chose 50 μM hemin for the following study which could induce erythroid differentiation in a short period of time but without causing K562 cell death.

We investigated the expression patterns of CRKL and miR‐429 during hemin‐induced erythroid differentiation of K562 cells. After treatment with 40 μM hemin, K562 cells showed significant increase in the number of benzidine‐positive cells in a time‐dependent manner. The benzidine‐positive rates of K562 cells induced by hemin for 0, 24 and 48 h were 2.5%, 26.8% and 34.9% respectively (Figure 2C). Meanwhile, the mRNA expression levels of erythroid differentiation markers γ‐globin, GPA, α‐globin and ε‐globin were also increased in K562 cells after treatment with hemin (Figure 2D), indicating hemin successfully induced the erythroid differentiation of K562 cells. Then we measured the expression level of miR‐429 and CRKL during hemin‐induced erythroid differentiation of K562 cells. qRT‐PCR showed that miR‐429 expression level was significantly upregulated by 43.5% (*p* = 0.0279) and 123.0% (*p* = 0.0011) in K562 cells following treatment with hemin for 24 and 48 h (Figure 2E), respectively. Meanwhile, western blotting (WB) assay showed that CRKL expression level was significantly downregulated by 28.7% (*p* = 0.0008) and 37.7% (*p* = 0.0072) in K562 cells following treatment with hemin for 24 and 48 h (Figure 2F), respectively. Taken together, miR‐429 expression was upregulated while CRKL expression was downregulated during hemin‐induced erythroid differentiation of K562 cells, indicating potential roles of miR‐429 and CRKL in erythroid differentiation.

### 3.3 miR‐429 deregulation affects the erythroid differentiation of K562 cells

**FIGURE 3:**
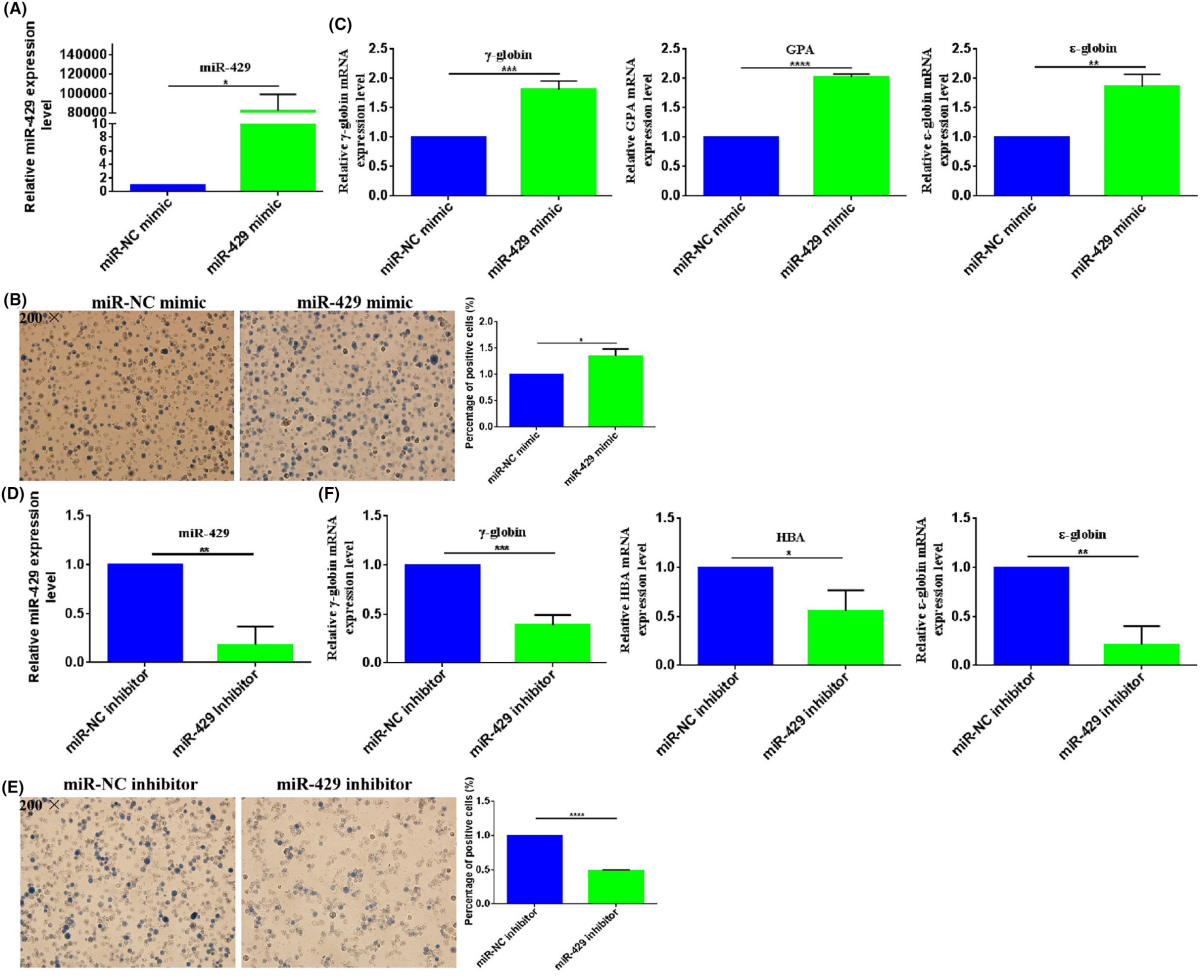
miR‐429 dysexpression affects the hemin‐induced erythroid differentiation of K562 cells. (A) miR‐429 was over‐expressed in K562 cells. miR‐429 mimic and miR‐NC mimic were transiently transfected into K562 cells. (B) The benzidine‐positive cells were counted in K562‐miR‐429 mimic and K562‐miR‐NC mimic cells. (C) γ‐globin, GPA and ε‐globin mRNA expression levels were detected in K562‐miR‐429 mimic and K562‐miR‐NC mimic cells by qRT‐PCR. (D) miR‐429 was downregulated in K562 cells. miR‐429 inhibitor and miR‐NC inhibitor were transiently transfected into K562 cells. (E) The benzidine‐positive cells were counted in K562‐miR‐429 inhibitor and K562‐miR‐NC inhibitor cells. (F) γ‐globin, HBA and ε‐globin mRNA expression levels were detected in K562‐miR‐429 inhibitor and K562‐miR‐NC inhibitor cells by qRT‐PCR. *, **, ***, ****Refer to *p* values <0.05, 0.01, 0.001, 0.0001.

To confirm its role in erythroid differentiation of CML, we measured the effects of miR‐429 upregulation and downregulation on the hemin‐induced erythroid differentiation of K562 cells. A synthetic double‐stranded miR‐429 mimic and single‐stranded miR‐429 inhibitor were transiently transfected into K562 cells to increase and decrease miR‐429 expression. qRT‐PCR showed miR‐429 expression level was increased by 82,036‐fold (*p* = 0.0209) in K562‐miR‐429 mimic cells compared with K562‐miR‐NC mimic (Figure 3A) and decreased by 81.7% (*p* = 0.0016) in K562‐miR‐429 inhibitor cells compared with K562‐miR‐NC inhibitor (Figure 3D), which provides control study for investigating miR‐429 dysexpression on the erythroid differentiation of CML cells.

miR‐429 dysexpression apparently affects the erythroid differentiation of K562 cells. miR‐429 expression level was positively correlated with the erythroid differentiation of K562 cells. miR‐429 overexpression promoted erythroid differentiation of K562 cells. K562‐miR‐429 mimic cells showed increased numbers of benzidine‐positive cells than K562‐miR‐NC mimic cells, the benzidine‐positive rates of K562‐miR‐429 mimic cells increased by 35.3% compared with K562‐miR‐NC mimic cells (*p* = 0.0106, Figure 3B). Meanwhile, the erythroid genes γ‐globin, GPA and ε‐globin mRNA expression levels also increased by 81.1% (*p* = 0.0007), 102.3% (*p* < 0.0001) and 85.7% (*p* = 0.0022) in K562‐miR‐429 mimic cells compared to K562‐miR‐NC mimic cells (Figure 3C). Our results showed that miR‐429 upregulation promoted erythroid differentiation of K562 cells.

Consistently, miR‐429 downregulation inhibited erythroid differentiation of K562 cells. K562‐miR‐429 inhibitor cells showed increased numbers of benzidine‐positive cells than K562‐miR‐NC inhibitor cells, the benzidine‐positive rates of K562‐miR‐429 inhibitor cells decreased by 51.4% compared with K562‐miR‐NC inhibitor cells (*p* = 0.0106, Figure 3E). Meanwhile, the erythroid genes γ‐globin, HBA and ε‐globin mRNA expression levels also decreased by 61.3% (*p* = 0.0005), 44.6% (*p* = 0.0226) and 78.9% (*p* = 0.002) in K562‐miR‐429 inhibitor cells compared to K562‐miR‐NC inhibitor cells (Figure 3F). Our results showed that miR‐429 downregulation inhibited erythroid differentiation of K562 cells. Clearly, miR‐429 significantly affected the erythroid differentiation of K562 cells.

### 3.4 CRKL deregulation affects the erythroid differentiation of K562 cells

**FIGURE 4:**
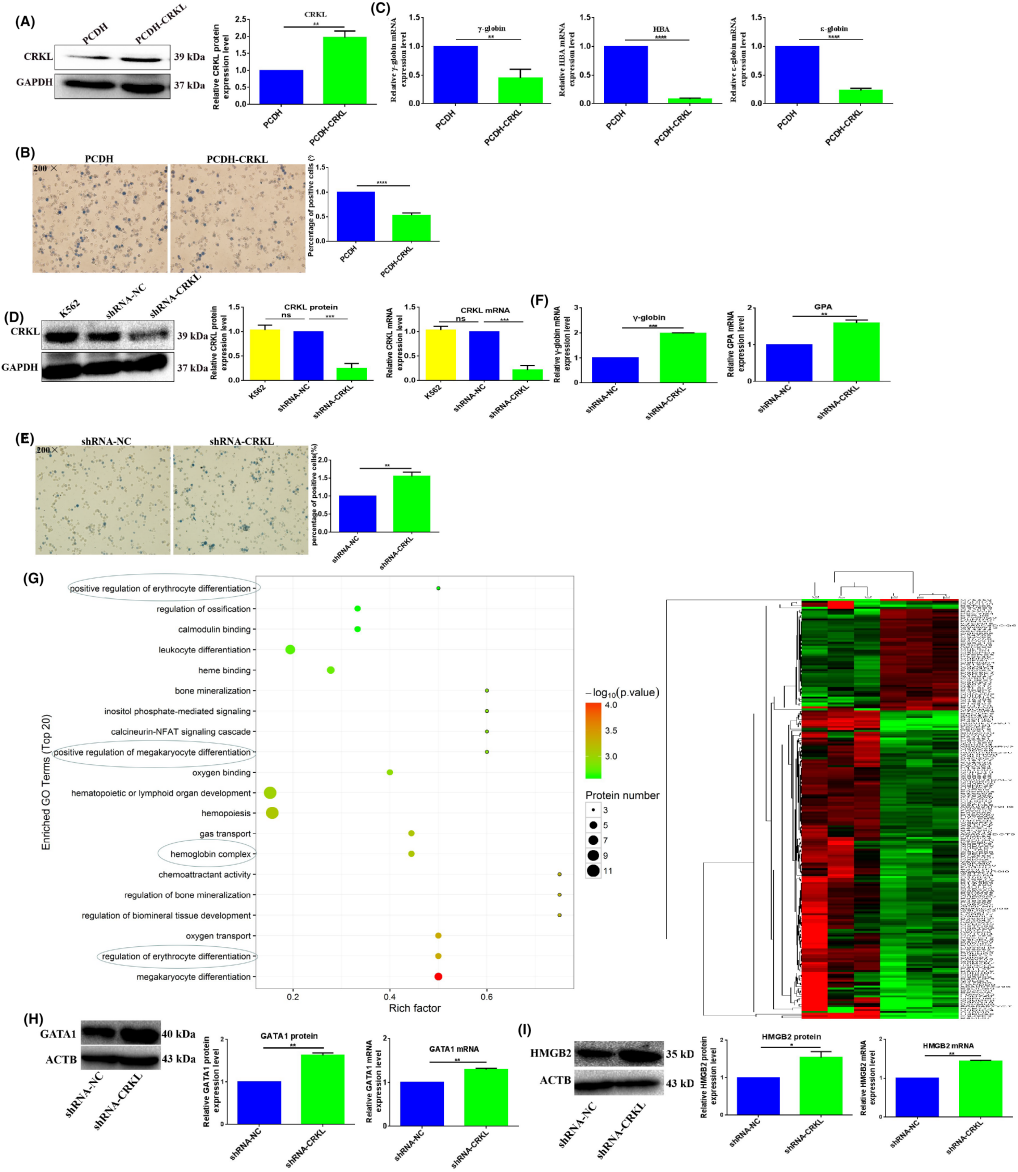
CT10 regulation of kinase‐like (CRKL) dysexpression affects the hemin‐induced erythroid differentiation of K562 cells. (A) CRKL was over‐expressed in K562 cells. (B) The benzidine‐positive cells were counted in K562‐PCDH‐CRKL and K562‐PCDH cells. (C) γ‐globin, HBA and ε‐globin mRNA expression levels were detected in K562‐PCDH‐CRKL and K562‐PCDH cells by qRT‐PCR. (D) CRKL was stably downregulated in K562 cells. (E) The benzidine‐positive cells were counted in K562‐shRNA‐CRKL and K562‐shRNA‐NC cells. (F) γ‐globin and GPA mRNA expression levels were detected in K562‐shRNA‐CRKL and K562‐shRNA‐NC cells by qRT‐PCR. (G) iTRAQ quantitative proteomic screened differentially expressed genes between K562‐shRNA‐CRKL and K562‐shRNA‐NC cells. (H and I) Western blotting assay (WB) and qRT‐PCR detected the protein and mRNA expression level of GATA‐1 and HMGB2 in K562‐shRNA‐CRKL and K562‐shRNA‐NC cells. *, **, ***, ****Refer to *p* values <0.05, 0.01, 0.001, 0.0001, ns refers to no statistical difference.

To gain insight into the role of CRKL in erythroid differentiation, we upregulated and downregulated its cellular expression level in K562 cells. First, we transiently transfected K562 cells with PCDH‐EF1‐MCS‐T2A‐Puro‐CRKL expression plasmid to overexpress CRKL. CRKL protein expression level was increased by 97.0% (*p* = 0.001, Figure 4A) in K562‐PCDH‐CRKL cells than in K562‐PCDH cells, which provides control study for the upregulation effect of CRKL on erythroid differentiation of K562 cells. Meanwhile, we successfully obtained the monoclonal cell line K562‐shRNA‐CRKL to knockdown endogenous CRKL expression. The CRKL protein and *CRKL* mRNA levels were decreased by 75.3% (*p* = 0.0002) and 78.9% (*p* = 0.0001) by RNAi (Figure 4D). The establishment of monoclonal K562‐shRNA‐CRKL cell with stable CRKL knockdown enabled the investigation of CRKL in erythroid differentiation of K562 cells, which provides a control study for the effect of CRKL downregulation on erythroid differentiation of K562 cells.

CRKL dysexpression apparently affects the erythroid differentiation of K562 cells. CRKL expression level was negatively correlated with the erythroid differentiation of K562 cells. CRKL overexpression inhibited erythroid differentiation of K562 cells. K562‐PCDH‐CRKL cells showed decreased numbers of benzidine‐positive cells than K562‐PCDH cells, the benzidine‐positive rates of K562‐PCDH‐CRKL cells decreased by 47.2% compared with K562‐PCDH cells (*p* < 0.0001, Figure 4B). Meanwhile, the erythroid genes γ‐globin, HBA and ε‐globin mRNA expression levels also decreased by 55.3% (*p* = 0.0034), 92.1% (*p* < 0.0001) and 76.8% (*p* < 0.0001) in K562‐PCDH‐CRKL cells compared to K562‐PCDH cells (Figure 4C). Our results showed that CRKL upregulation inhibited erythroid differentiation of K562 cells.

Consistently, CRKL downregulation promoted erythroid differentiation of K562 cells. K562‐shRNA‐CRKL cells showed increased numbers of benzidine‐positive cells than K562‐shRNA‐NC cells. The benzidine‐positive rates of K562‐shRNA‐CRKL cells increased by 54.5% compared with K562‐shRNA‐NC cells (*p* = 0.0013, Figure 4E). Meanwhile, the erythroid genes γ‐globin and GPA mRNA expression levels also increased by 96.9% (*p* = 0.0006) and 59.4% (*p* = 0.0096) in K562‐shRNA‐CRKL compared to K562‐shRNA‐NC cells (Figure 4F). Our results showed that CRKL downregulation promoted erythroid differentiation of K562 cells.

We further screened the differentially expressed genes between K562‐shRNA‐CRKL and K562‐shRNA‐NC cells by gene microarray (Affymetrix GeneChip Human Transcriptome Array 2.0). A total of 549 mRNAs were identified as up‐ or downregulated over 1.5‐fold in K562‐shRNA‐CRKL cells compared with K562‐shRNA‐NC cells. Among these targeting genes, we focused on the molecules associated with erythroid differentiation. We found that haemoglobin (HB) molecules HBD, HBA1, HBA2 and HBZ were upregulated 1.6‐, 2.2‐, 2.3‐ and 2.5‐fold in K562‐shRNA‐CRKL cells compared to K562‐shRNA‐NC cells (Table S2), meanwhile, the erythroid‐associated molecules SPTA1, SPTB, TFRC, PKLR, EPB41L3, AHSP, RHD and RHCE were upregulated 1.5‐, 1.6‐, 1.8‐, 1.8, 1.9‐, 2.2‐, 1.9‐ and 2.1‐fold in K562‐shRNA‐CRKL cells compared to K562‐shRNA‐NC cells (Table S2).

Moreover, we screened the differentially expressed proteins between K562‐shRNA‐CRKL and K562‐shRNA‐NC cells by iTRAQ quantitative proteomic analysis. A total of 215 proteins were identified as up‐ or downregulated over 1.2‐fold (*p* < 0.05) in K562‐shRNA‐CRKL cells compared with K562‐shRNA‐NC cells. Among these differently expression proteins, 53 proteins were upregulated and 162 proteins were downregulated in K562‐shRNA‐CRKL cells compared to K562‐shRNA‐NC cells, these proteins were clustered as shown in Figure 4G. Gene ontology analysis was performed on the differentially expressed proteins, these differentially expressed proteins were related to positive regulation of erythrocyte differentiation, leukocyte differentiation, positive regulation of megakaryocyte differentiation, haemoglobin complex, regulation of erythrocyte differentiation and megakaryocyte differentiation, indicating CRKL deregulation was associated with differentiation of K562 cells and further validating CRKL downregulation promoted erythrocyte differentiation. Among these targeting proteins, we also focused on the molecules associated with erythroid differentiation. Consistently with microarray results, we found that haemoglobin molecules HBE1, HBD, HBZ, HBG1, erythroid‐specific transcription factor GATA‐1, HMGB2 and PKLR were upregulated 1.2‐, 1.3‐, 1.2‐, 1.2‐, 1.3‐, 1.2‐ and 1.2‐fold in K562‐shRNA‐CRKL cells compared to K562‐shRNA‐NC cells (Table S3), respectively. We detected the expression levels of GATA‐1 and HMGB2 by WB and qRT‐PCR to validate the proteomic analysis results. The protein and mRNA expression levels of GATA‐1 were upregulated 63.0% (*p* = 0.004) and 29.5% (*p* = 0.0041) in K562‐shRNA‐CRKL cells compared to K562‐shRNA‐NC cells (Figure 4H), respectively, meanwhile, the protein and mRNA expression levels of HMGB2 were upregulated 54.0% (*p* = 0.0391) and 44.1% (*p* = 0.0012) in K562‐shRNA‐CRKL cells compared to K562‐shRNA‐NC cells (Figure 4I), respectively. The expression profile was consistent with proteomic analysis results, indicating the proteomic analysis results were believable.

Our results indicated that CRKL downregulation promoted haemoglobin molecules expression, which resulted in erythroid differentiation of K562 cells. Taken together, the gene microarray and iTRAQ quantitative proteomic analysis further confirmed that CRKL downregulation promoted erythroid differentiation of K562 cells. Clearly, CRKL significantly affected the erythroid differentiation of K562 cells. Our results indicated that CRKL acted as a erythroid differentiation inhibitor in CML by promoting the aggressiveness of leukaemia cells.

### 3.5 miR‐429 regulates erythroid differentiation of K562 cells by downregulating CRKL through binding with *CRKL* ‐3′‐UTR

**FIGURE 5:**
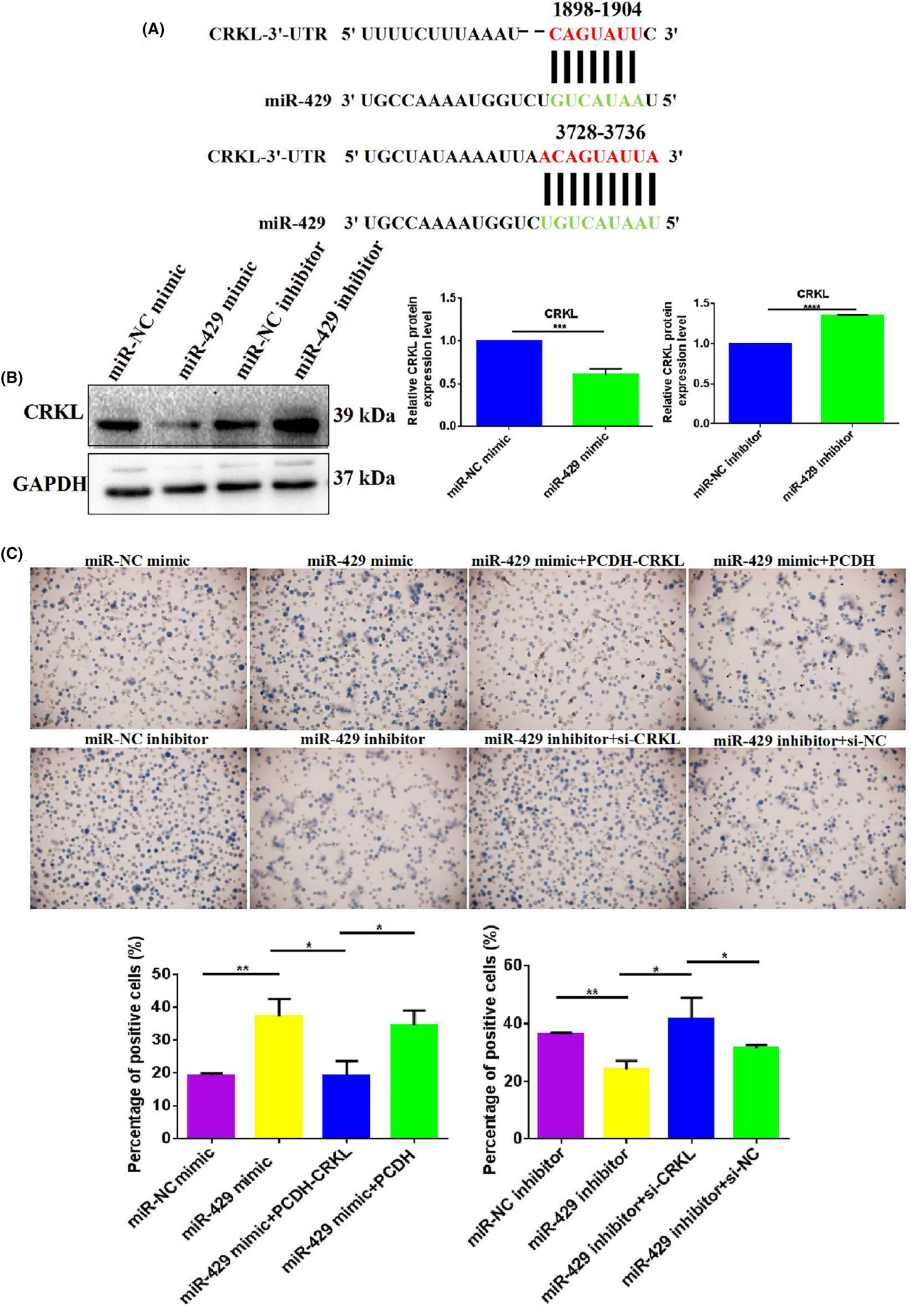
miR‐429 promotes chronic myeloid leukaemia (CML) erythropoiesis by downregulating CT10 regulation of kinase‐like (CRKL) expression through directly targeting to *CRKL*‐3′‐untranslated region (UTR). (A) Putative binding sites for miR‐429 at *CRKL*‐3′‐UTR. (B) miR‐429 overexpression decreased endogenous CRKL expression level in K562 cells and miR‐429 suppression increased endogenous CRKL expression level in K562 cells. (C) CRKL overexpression or suppression neutralized miR‐429 overexpression‐mediated promotion or reversed miR‐429 knockdown‐mediated inhibition on erythropoiesis of K562 cells. *, **, ***, ****Refer to *p* values <0.05, 0.01, 0.001, 0.0001, ns refers to no statistical difference.

Bioinformatics analysis indicated that CRKL was a potential target gene of miR‐429, with two putative binding sites of 1898‐1904 and 3728‐3735 at *CRKL*‐3′‐UTR region for miR‐429 (Figure 5A) and we have previously reported that CRKL was a direct downstream target of miR‐429 via directly binding to site 2 in its 3′‐UTR by luciferase reporter assay.^38^, ^39^ We also observed a significant negative correlation between the expression levels of miR‐429 and CRKL in CML patients, to further confirm the negative correlation between miR‐429 and CRKL, we detected the effect of miR‐429 deregulation on the expression levels of CRKL in K562 cells, miR‐429 overexpression decreased endogenous CRKL expression level (Figure 5B). In comparison with K562‐miR‐NC mimic cells, CRKL expression level was decreased by 39.3% (*p* = 0.0006) in K562‐miR‐429 mimic cells. Consistently, miR‐429 downregulation increased endogenous CRKL expression level (Figure 5B). In comparison with K562‐miR‐NC inhibitor cells, miR‐429 expression level was increased by 34.7% (*p* < 0.0001) in K562‐miR‐429 inhibitor cells. Our results further demonstrated miR‐429 expression was negatively correlated with CRKL expression in CML cells, indicating miR‐429 negatively regulated CRKL expression.

Is CRKL a direct functional mediator of miR‐429‐promoted hemin‐induced erythroid differentiation for K562 cells? Could the in vitro phenotypes associated with miR‐429 deregulation be reversed by opposite CRKL expression? We performed a ‘rescue’ experiment by co‐transfecting miR‐429 mimic and PCDH‐CRKL vectors or miR‐429 inhibitor and si‐CRKL into K562 cells. Exogenous CRKL overexpression markedly counteracted the promotion effect of miR‐429 on the hemin‐induced erythroid differentiation of K562 cells (Figure 5C), meanwhile, endogenous CRKL kncokdown significantly reversed the miR‐429 downregulation‐mediated inhibition effect on the hemin‐induced K562 cell erythroid differentiation (Figure 5C). These aforementioned results collectively demonstrated that CRKL was a functional effector of miR‐429 mediated erythropoiesis‐promoting effect, and miR‐429 promoted CML erythropoiesis by downregulating CRKL expression.

### 3.6 miR‐429‐CRKL axis mediates erythroid differentiation of K562 cells via Raf/MEK/ERK pathway

The underlying molecular mechanism of miR‐429‐CRKL axis in erythroid differentiation is unknown. Current work linked miR‐429‐mediated action on erythroid differentiation of K562 via Raf/MEK/ERK by targeting CRKL. As the Raf/MEK/ERK pathway was involved in erythropoiesis, we hypothesized that miR‐429‐CRKL axis affected K562 cell differentiation via the Raf/MEK/ERK pathway.

**FIGURE 6:**
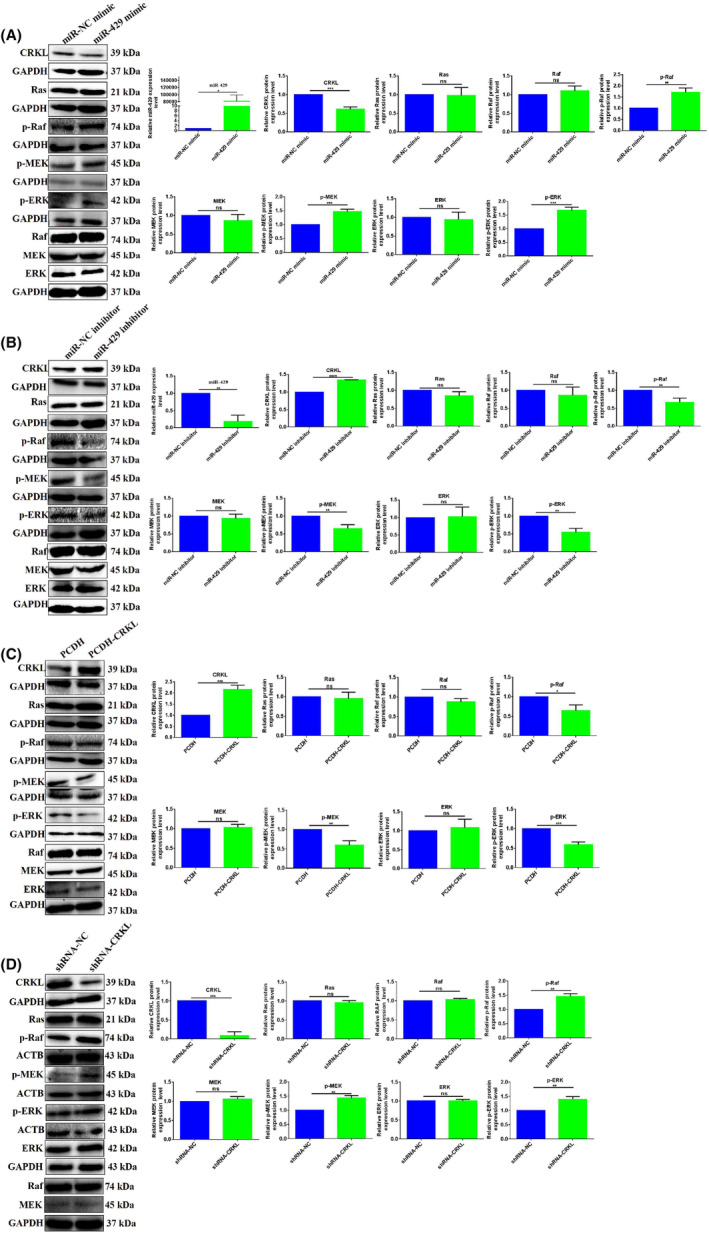
The effects of miR‐429 and CT10 regulation of kinase‐like (CRKL) dysexpression on Raf/MEK/ERK pathway in K562 cells. (A) miR‐429 overexpression activated the Raf/MEK/ERK pathway. (B) miR‐429 knockdown inactivated the Raf/MEK/ERK pathway. (C) CRKL overexpression inactivated the Raf/MEK/ERK pathway. (D) CRKL knockdown activated the Raf/MEK/ERK pathway. *, **, ***Refer to *p* values <0.05, 0.01, 0.001, ns refers to no statistical difference.

We found miR‐429 overexpression or CRKL knockdown resulted in the similar trend on Raf/MEK/ERK pathway. miR‐429 overexpression or CRKL knockdown activated the Raf/MEK/ERK pathway. miR‐429 overexpression decreased the protein expression level of CRKL by 39.3% (*p* = 0.0006) and increased the protein expression levels of p‐Raf, p‐MEK and p‐ERK by 71.3% (*p* = 0.0034), 46.7% (*p* = 0.0007), 67.7.6% (*p* = 0.0004), respectively (Figure 6A), meanwhile, CRKL knockdown decreased the protein expression level of CRKL by 91.2% (*p* = 0.0001) and increased the protein expression levels of p‐Raf, p‐MEK and p‐ERK by 45.3% (*p* = 0.0013), 42.6% (*p* = 0.0014) and 39.6% (*p* = 0.0019) respectively (Figure 6D). Consistently, miR‐429 knockdown or CRKL overexpression inactivated the Raf/MEK/ERK pathway. miR‐429 knockdown increased the protein expression level of CRKL by 34.7% (*p* < 0.0001) and decreased the protein expression levels of p‐Raf, p‐MEK and p‐ERK by 33.0% (*p* = 0.0071), 35.3% (*p* = 0.0045) and 46.3% (*p* = 0.0025) respectively (Figure 6B), meanwhile, CRKL overexpression increased the protein expression level of CRKL by 116.3% (*p* = 0.0004) and decreased the protein expression levels of p‐Raf, p‐MEK and p‐ERK by 35.7% (*p* = 0.0147), 40.7% (*p* = 0.0039) and 41.0% (*p* = 0.0005) respectively (Figure 6C). No changes were observed for Ras, Raf, MEK and ERK (Figure 6). The results indicated that miR‐429‐CRKL axis mediated the erythroid differentiation of CML cells via Raf/MEK/ERK signalling pathway.

**FIGURE 7:**
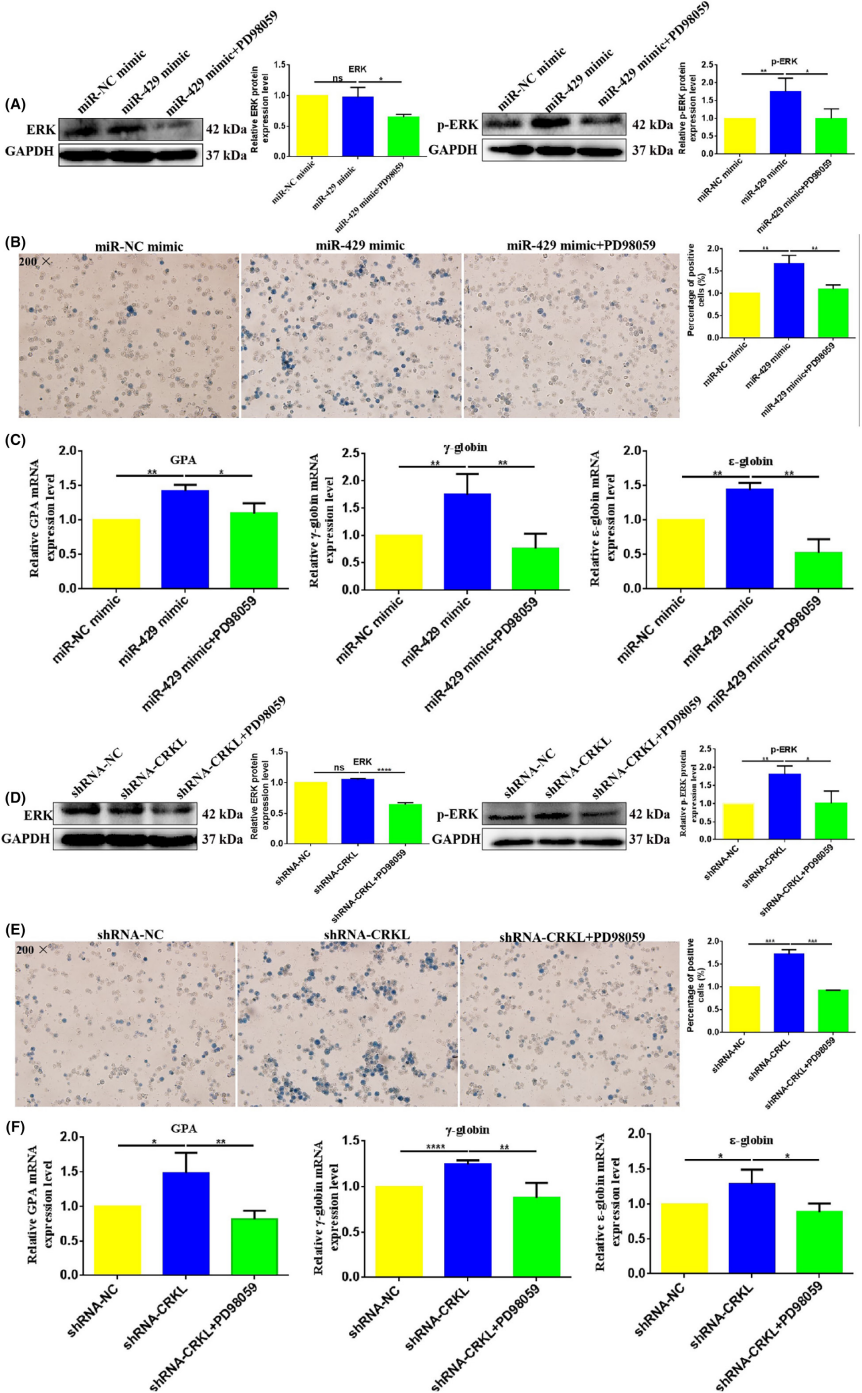
miR‐429‐CT10 regulation of kinase‐like (CRKL) axis mediates the erythroid differentiation of chronic myeloid leukaemia (CML) cells via Raf/MEK/ERK signalling pathway. (A) Western blotting (WB) assay of ERK and p‐ERK expression level changes in K562‐miR‐429 mimic cells after treatment with PD98059. (B) The benzidine‐positive cell was decreased in K562‐K562‐miR‐429 mimic cells after treatment with PD98059. (C) The erythroid genes GPA, γ‐globin and ε‐globin mRNA expression levels decreased in K562‐K562‐miR‐429 mimic cells after treatment with PD98059. (D) WB assay of ERK and p‐ERK expression level changes in K562‐shRNA‐CRKL cells after treatment with PD98059. (E) The benzidine‐positive cell was decreased in K562‐shRNA‐CRKL cells after treatment with PD98059. (F) The erythroid genes GPA, γ‐globin and ε‐globin mRNA expression levels decreased in K562‐shRNA‐CRKL cells after treatment with PD98059. *, **, ***, ****Refer to *p* values <0.05, 0.01, 0.001, 0.0001, ns refers to no statistical difference.

The linkage of the Raf/MEK/ERK pathway to miR‐429‐CRKL axis‐mediated erythroid differentiation was validated using PD98059. The treatment of K562‐miR‐429 mimic (Figure 7A) and K562‐shRNA‐CRKL (Figure 7D) cells with PD98059 resulted in reduced protein expression levels of ERK and p‐ERK, indicating PD98059 successfully blocked the Raf/MEK/ERK pathway. Furthermore, we detected the erythroid differentiation change after blocking the Raf/MEK/ERK pathway. The erythroid differentiation was inhibited after blocking the Raf/MEK/ERK pathway in K562‐miR‐429 mimic and K562‐shRNA‐CRKL cells. The benzidine‐positive cell was decreased by 34.5% (*p* = 0.0088) and 55.5% (*p* = 0.0002) in K562‐miR‐429 mimic (Figure 7B) and K562‐shRNA‐CRKL (Figure 7E) cells after blocking the Raf/MEK/ERK pathway. Meanwhile, the erythroid genes GPA, γ‐globin and ε‐globin mRNA expression levels were also decreased by 23.1% (*p* = 0.0286), 56.2% (*p* = 0.0015), 63.6% (*p* = 0.0022) and 44.5% (*p* = 0.0059), 46.0% (*p* = 0.0052), 30.9% (*p* = 0.0172) in K562‐miR‐429 mimic (Figure 7C) and K562‐shRNA‐CRKL (Figure 7F) cells after blocking the Raf/MEK/ERK pathway. These results indicated that miR‐429‐CRKL axis regulated erythroid differentiation of CML cells by mediating Raf/MEK/ERK pathway.

### 3.7 CRKII has no effect on erythroid differentiation of K562 cells

**FIGURE 8:**
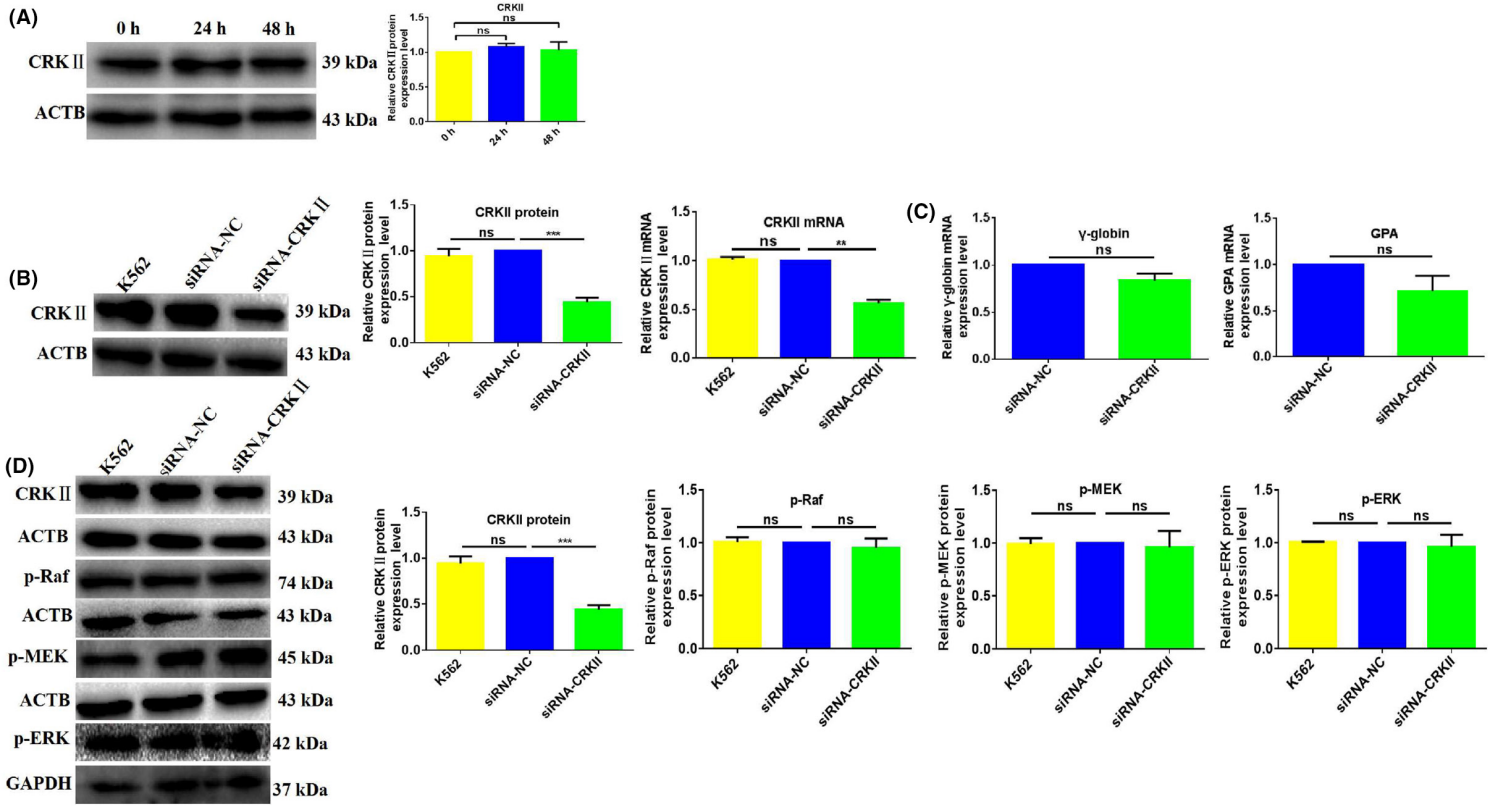
CT10 regulation of kinase II (CRKII) downregulation has no effect on erythroid differentiation of K562 cells. (A) CRKII protein expression level was measured in hemin‐induced K562 cells by western blotting (WB) assay. (B) CRKII downregulated in K562 cells by siCRKII transient transfection interference. (C) γ‐globin and GPA mRNA expression levels were detected in K562‐siRNA‐CRKII and K562‐siRNA‐NC cells by qRT‐PCR. (D) The effect of CRKII downregulation on the Raf/MEK/ERK signalling pathway. **, ***Refers to *p* value <0.01, 0.001, ns refers to no statistical difference.

As members of the CRK adaptor protein family, CRKII and CRKL are highly similar in sequence and both possess tyrosine phosphorylation sites that can be phosphorylated by BCR‐ABL to activate signalling pathways. So we investigated the effect of CRKII on erythroid differentiation. First, we investigated the expression pattern of CRKII during hemin‐induced erythroid differentiation of K562 cells. No obvious expression level change was observed for CRKII during erythroid differentiation of K562 cells. CRKII protein expression level was only slightly upregulated by 7.6% (*p* = 0.058) and 3.1% (*p* = 0.6724) in K562 cells following treatment with hemin for 24 and 48 h (Figure 8A), respectively, indicating CRKII might not be involved in erythroid differentiation of K562 cells.

CRKII may be unimportance for erythroid differentiation, to confirm the effect of CRKII on erythroid differentiation, we transiently transfected K562 cells with siCRKII to knockdown CRKII. CRKII protein and mRNA levels were decreased by 50.1% (*p* = 0.0007) and 43.5% (*p* = 0.0059) in K562‐siRNA‐CRKIIcompared with K562‐siRNA‐NC cells, respectively (Figure 8B), providing a control study for the downregulation effect of CRKII on erythroid differentiation of K562 cell. qRT‐PCR detected the expression level changes of erythroid genes after CRKII knockdown, there were no obvious changes in γ‐globin (*p* = 0.0882) and GPA (*p* = 0.1333) mRNA expression levels between K562‐siRNA‐CRKIIand K562‐siRNA‐NC cells (Figure 8C).

Meanwhile, CRKII downregulation did not affect the Raf/MEK/ERK pathway. We measured the expression level changes of p‐Raf, p‐MEK and p‐ERK after CRKII knockdown by WB. No changes were observed for p‐Raf, p‐MEK and p‐ERK (Figure 8D). Clearly, our results further demonstrate that CRKII had no effect on erythroid differentiation of K562 cells. Collectively, CRKL could inhibit erythroid differentiation of K562 cells.

## 4 DISCUSSION

Haematopoiesis is a highly and precisely regulated multistage process by which all of the different cell lineages (erythroid cells, lymphocytes and myeloid cells) that form the immune and blood systems originate from pluripotent stem cells.^43^, ^44^ Erythropoiesis happens in human red BM after kidneys responses to low levels of oxygen by releasing erythropoietin.^45^ Erythropoiesis is a multistep cellular course by which a primitive multipotent HSC experiences a series of differentiations resulting in production of erythroid lineage, undergoing erythroid progenitors (colony‐forming unit erythroid [CFU‐E] and burst‐forming unit erythroid [BFU‐E]), normoblasts, proerythroblasts, early basophilic erythroblasts, late basophilic erythroblasts, polychromatic erythroblasts, orthochromatic erythroblasts, reticulocytes, ultimately differentiating to mature erythrocytes.^7^, ^8^ The dynamic process is mediated by a balance of intrinsic and extrinsic factors, containing transcription factors, growth factors and miRNAs, and destruction of the dynamic process leads to CML. Tyrosine kinase inhibitors (TKIs) targeting BCR‐ABL for CML therapy have effectively improved the survival of CML patients; however, about 20% of CML patients have not been benefited from TKIs treatment, commonly due to TKIs resistance which lead to disease relapse and progression.^46^, ^47^, ^48^ Therefore, it is urgent to seek more efficient therapeutic strategies to overcome the problem. Deeper study of the molecular mechanisms governing the development, progression and differentiation of CML can lead to finding novel therapeutic targets and improving the therapy effects for CML patients.

We screened the differentially expressed ETV6 gene between K562‐shRNA‐CRKL and K562‐shRNA‐NC cells by iTRAQ quantitative proteomic and gene microarray assays. ETV6 is originally involvement in chromosomal translocation linked with hematologic and various cancers, a lot of chromosomal translocation oncogenes have been identified, as a transcriptional repressor, ETV6 is biologically important in embryonic development and haematopoietic regulation. Moreover, we found ETV6 was overexpressed in CML patients compared with normal samples, ETV6 could regulate hemin‐induced erythroid differentiation of K562 cell through the Raf/MEK/ERK pathway.^49^ Furthermore, ETV6 (Tel) is known to forms fusion protein by chromosomal translocations with ABL. CRKL is a substrate protein for ABL, and the Tel‐ABL fusion protein can form complexes with CRKL in leukaemia, we have also shown that ETV6 directly binds to CRKL by Co‐IP assay further indicated the direct interaction between ETV6 and CRKL.^41^ Meanwhile, bioinformatics combined with the ChIP assay revealed that by directly binding to the DNA promoter region GGAGGAAGCA at the 696‐705, ETV6 reversely mediated the expression of miR‐429 in hepatocarcinoma cells, miR‐429 also negatively regulates CRKL expression by selectively targeting CRKL‐3′‐UTR.^41^ Therefore, we spectulated miR‐429 regulates erythroid differentiation by directly targeting CRL.

The current study has identified a miR‐429‐CRKL signalling axis that played an important role in CML, and provided a comprehensive mechanism for the tumorigenesis and erythroid differentiation of CML in which miR‐429 negatively regulates CRKL expression by targeting *CRKL*‐3′‐UTR. The information gained from this research has important clinical implications for patients with CML as well as other cancer types associated with elevated CRKL expression and decreased miR‐429 expression, and may also have clinical impact on other diseases with dysregulated expression of CRKL and miR‐429. In the current work, we only collected 34 CML primary patient BM samples, 6 CML CR patient BM samples and 13 healthy subject normal PB samples, the sample size is relatively small, and the amounts of the mononuclear BM and peripheral blood cells were separated from the CML patients and healthy subject normal samples were very small, the amount of the samples is not enough to detect the expression level of CRKL by WB, so we studied the expression pattern of CRKL and miR‐429 in CML by qRT‐PCR, but our results are reliable, because we also detected the expression patterns and the correlations of CRKL and miR‐429 in ccRCC patients' tumorous tissues and matched paracancerous nontumoral renal tissues,^41^ and HCC patients' tumorous tissues and matched paracancerous nontumoral liver tissues,^40^ the expression patterns and the correlations of CRKL and miR‐429 in CML consistent with those in ccRCC and HCC. In the current work, we mainly investigated the potential role of miR‐429‐CRKL axis in erythroid differentiation of CML.

CRKL deregulation is linked to the development and progression of a variety of cancers.^27^, ^28^, ^29^ As we summarized in our review,^26^ abnormal CRKL expression is associated with HCC, RCC, gastric cancer, glioblastoma multiforme, bladder cancer, lung cancer, colon cancer, ovarian cancer, leukaemia, breast cancer, head and neck cancer, rhabdomyosarcoma and neuroblastoma. It is of promise as an indicator for cancer development, invasion and metastasis as well as an attractive target for the diagnosis and prognosis of cancer. CRKL is a major tyrosine‐phosphorylated protein in CML cells, pCRKL plays a special role in CML pathogenesis, and the constitutive phosphorylation of CRKL is unique to CML, which makes it a useful target for therapeutic intervention.^50^, ^51^, ^52^ We previous reported that CRKL is upregulated in HCC and ccRCC, and associated with proliferation, migration and invasion of HCC and ccRCC.^40^, ^41^, ^53^, ^54^, ^55^ However, the exact role of CRKL in CML is unknown. Our current work showed that CRKL expression potentially promotes the clinical development and progression of CML patients and enhances CML cell aggressiveness. CRKL was universally overexpressed in CML primary patient samples compared with normal samples (Figure 1A). Interestingly, CRKL expression level was lower in CML CR patient samples than in corresponding CML primary patient samples (Figure 1B). Our results indicate that CRKL is a tumour promoter playing a vital role in the development and progression of CML. To the best of our knowledge, this work is the first reporting the expression pattern of CRKL in CML patients, CR patients and normal samples.

It is known that the CRK family plays important roles in the regulation of cell differentiation. v‐CRK overexpression can increase rat pheochromocytoma PC12 cell differentiation,^29^ and both SH2 and SH3 domains of the CRK protein are required for PC12 cell expression.^31^ Moreover, CRKII enhances osteoclast differentiation by activating Rac1, the overexpression of CRKII and CRKL significantly enhance RANKL‐induced osteoclast differentiation, and the downregulation of CRKII and CRKL synergistically decrease RANKL‐induced osteoclast differentiation.^32^ The effect of CRKL on leukaemia cell differentiation has not been reported, in our study we investigated the potential role of CRKL in erythroid differentiation of K562 cells. Hemin is an iron‐containing porphyrin which is involved in oxygen delivery and used to treat acute porphyria and thalassemia intermedia, and is also a relatively strong inducer for heme biosynthesis of K562 cell erythroid differentiation.^56^ Using hemin‐induced K562 cell as a model, we found that CRKL expression level was downregulated in hemin‐induced erythroid differentiation of K562 cells (Figure 2F), indicating CRKL might play an important role in erythroid differentiation of K562 cells. In order to verify the hypothesis, we successfully constructed CRKL stably downregulated monoclonal cell line to investigate the effect of endogenous CRKL on erythroid differentiation. We found that CRKL downregulation promoted hemin‐induced erythroid differentiation of K562 cells with more benzidine‐positive cells (Figure 4E), higher mRNA expression levels of γ‐globin and GPA (Figure 4F) and higher protein expression levels of GATA‐1, HMGB2 (Figure 4H,I). Furthermore, we screened the differentially expressed molecules between K562‐shRNA‐CRKL and K562‐shRNA‐NC cells using gene microarray and iTRAQ quantitative proteomic analysis, results showed haemoglobin molecules HBD, HBA1, HBA2, HBZ, HBE1 and HBG1 were upregulated in K562‐shRNA‐CRKL than in K562‐shRNA‐NC cells (Figure 4G, Tables S2 and S3). Consistently, CRKL overexpression inhibited hemin‐induced erythroid differentiation of K562 cells with fewer benzidine‐positive cells (Figure 4B) and lower mRNA expression levels of γ‐globin, HBA and ε‐globin (Figure 5C). Our results demonstrated that CRKL negatively regulated the erythroid differentiation, which as an inhibitor in the erythroid differentiation of CML.

miR‐429 is a member of the miR‐200 family, miR‐429 abnormal expression is linked to a variety of cancers.^57^ It shows suppression or promotion effects on tumour development, metastasis, apoptosis and drug‐resistance depending on the tumour type and subtype.^39^ It is a potential indicator for the diagnosis, treatment and prognosis of certaincancers.^39^ We previous reported that miR‐429 is downregulated in HCC and ccRCC, and associated with migration and invasion of HCC and ccRCC.^40^, ^41^ However, the exact role of miR‐429 in CML is unknown. Our current work showed that the deficiency of miR‐429 potentially promotes the clinical development and progression of CML patients and enhances CML cell aggressiveness. miR‐429 was downregulated in CML primary patient samples compared with normal samples (Figure 1C). Interestingly, miR‐429 expression level was higher in CML CR patient samples than in corresponding CML primary patient samples (Figure 1D). Our results indicate that miR‐429 plays a suppressive role in the development and progression of CML. To the best of our knowledge, this work is the first reporting the expression pattern of miR‐429 in CML patients, CR patients and normal samples.

The effect of miR‐429 on leukaemia cell differentiation has not been reported, in our study we further investigated the potential role of miR‐429 in erythroid differentiation of K562 cells. We found that miR‐429 expression level was upregulated in hemin‐induced erythroid differentiation of K562 cells (Figure 2E), indicating miR‐429 might play an important role in erythroid differentiation of K562 cells. We first investigated the effects of miR‐429 deregulation on hemin‐induced erythroid differentiation of K562 cells. The miR‐429 overexpression remarkably promoted the erythroid differentiation of K562 cells (Figure 3B,C), while, its knockdown significantly inhibited the erythroid differentiation of K562 cells (Figure 4F,G). Our results demonstrated that miR‐429 positively regulated the erythroid differentiation, which as a promotor in the erythroid differentiation of CML.

Previously, we reported that miR‐429 negatively regulates CRKL expression in HCC and ccRCC cells by selectively targeting its 3′‐UTR at the 3728‐3735 bp site and the upregulation of CRKL negatively correlated with miR‐429 deficiency potentially promoted the development and progression of HCC/ccRCC patients and the aggressiveness of HCC/ccRCC cells.^40^, ^41^ The current work also established the association of CRKL with miR‐429 in CML. CRKL expression level was negatively correlated with miR‐429 in CML patients (Figure 1E), a negative correlation was also established for CRKL upregulation with miR‐429 downregulation in three pairs of CML primary and CR patient samples (Figure 1F). Meanwhile, we further demonstrated that miR‐429 negatively regulated CRKL expression in K562 cells. miR‐429 overexpression and knockdown could decrease and increase (Figure 5B) the expression levels of CRKL in 562 cells. Are the miR‐429‐mediated influences on K562 properties linked to CRKL dysexpression? Is CRKL a direct functional mediator of miR‐429 on erythroid differentiation of K562 cells? The ‘rescue’ experiment found CRKL overexpression counteracted the promotion effect of miR‐429 upregulation on the hemin‐induced erythroid differentiation of K562 cells (Figure 5C), meanwhile, CRKL kncokdown reversed the inhibition effect of miR‐429 downregulation on the hemin‐induced erythroid differentiation of K562 cells (Figure 5C). The above results once again proved that CRKL is a functionally relevant effector of miR‐429 mediated erythroid differentiation effect, and miR‐429 promoted erythroid differentiation of K562 cells by directly targeting *CRKL*‐3′‐UTR to downregulate its expression.

miR‐429‐CRKL axis regulated HCC and ccRCC malignant progression through Raf/MEK/ERK and SOS1/MEK/ERK/MMP2/MMP9 pathways,^40^, ^41^ previously, we also found that CRKL regulated proliferation, migration and invasion of K562 cells through PI3K‐Akt/mTOR and p130CAS/CRKL/Dock180/RAC1 pathways (unpublished). The Raf/MEK/ERK signalling pathway is involved in erythropoiesis which mainly promotes growth, differentiation and prevents apoptosis of haematopoietic cells.^58^, ^59^, ^60^ MASL1 could induce erythroid differentiation in CD34^+^ cells through the Raf/MEK/ERK signalling pathway.^61^ G protein expression levels increased and ERK activated during hemin‐induced erythroid differentiation of K562 cells.^62^ Inhibition of the MEK/ERK signalling pathway promoted erythroid differentiation and reduced HSCs engraftment in ex vivo expanded HSC.^63^ In the current work, we explored the molecular mechanism of miR‐429‐CRKL axis on erythroid differentiation. Our results showed that miR‐429 and CRKL affected the expression levels of Raf/MEK/ERK pathway‐related molecules (Figure 6), we speculated miR‐429 and CRKL might mediate erythroid differentiation of K562 cells by regulating the Raf/MEK/ERK pathway. We validated the potential involvement of Raf/MEK/ERK using a specific ERK inhibitor PD98059. The benzidine‐positive cells and erythroid genes GPA, γ‐globin and ε‐globin mRNA expression levels decreased after blocking the Raf/MEK/ERK pathway (Figure 7). The results indicated that miR‐429‐CRKL axis regulated erythroid differentiation of K562 cells via Raf/MEK/ERK pathway.

CRKII and CRKL share a high degree of homology within their functional domains, CRKL is the major tyrosine‐phosphorylated protein in BCR‐ABL‐driven CML patient neutrophils.^64^ The preferential binding of BCR‐ABL to CRKL, even in the presence of CRKII,^65^ suggests disparity in interaction properties and differential regulation of CRK proteins by BCR‐ABL or ABL tyrosine kinases. These finds imply that CRKII and CRKL may play different role in CML, so in this work we also investigated the exact effect of CRKII on erythropoiesis of CML. The current study illustrated for the first time that CRKL but not CRKII inhibits erythroid differentiation via the inactivating Raf/MEK/ERK pathway. The expression level of CRKII was not changed in hemin‐induced erythroid differentiation of K562 cells (Figure 8A) and CRKII downregulation did not affect erythroid differentiation of K562 cells (Figure 8B,C). Moreover, CRKII downregulation could not affect the Raf/MEK/ERK pathway (Figure 8D), further indicating CRKII had no effect on erythroid differentiation of K562 cells. Collectively, CRKII is not associated with erythroid differentiation of K562 cells. Although CRKII and CRKL have a high degree of similarity in sequence, the two isoforms vary in ligand affinities and specificity, and the three‐dimensional structures of CRKII and CRKL differ to engage key signalling partners.^22^, ^29^ The respective knockout mice have distinct phenotypes but both proteins are required for embryonic development.^66^, ^67^ So CRKII and CRKL might function differently in leukemogenesis and erythropoiesis of CML, which deserves more attention to understand the differences between the two CRK adapter proteins. Our results are also consistent with the previous report that CRKL expression level is the highest in adult haematopoietic tissues and low in epithelial tissues, whereas CRKII exhibits the highest expression in the brain, lung, kidney and low expression in BM.^68^

**FIGURE 9:**
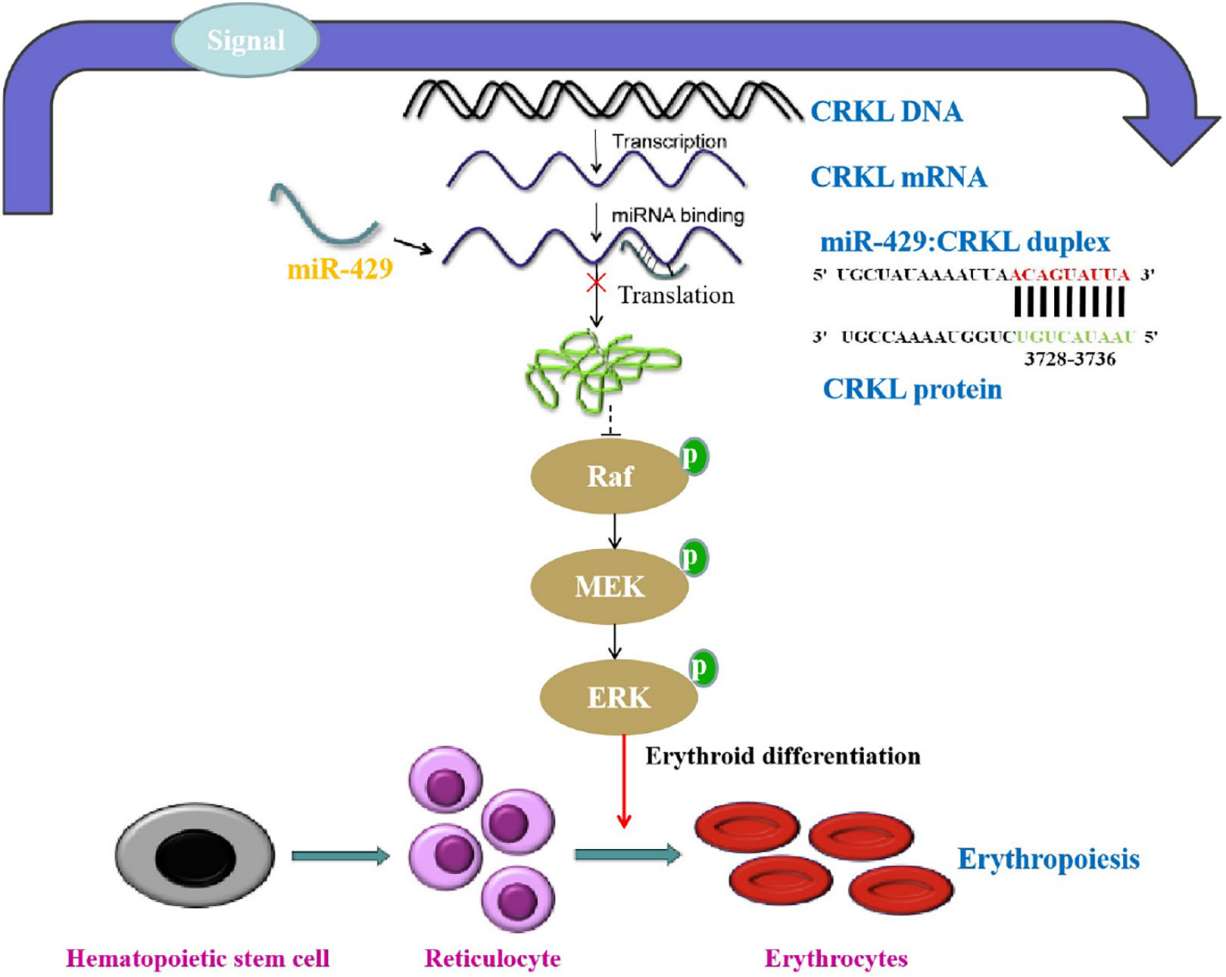
The schematic regulation mechanism of miR‐429‐ CT10 regulation of kinase‐like (CRKL) axis on erythropoiesis. miR‐429 negatively regulate CRKL expression by selectively targeting *CRKL*‐3′‐untranslated region (UTR), then promotes erythropoiesis through Raf/MEK/ERK pathway.

Taken together, our results showed that miR‐429‐CRKL axis contributes to erythropoiesis of CML by regulating Ras/Raf/ERK pathway. As illustrated in Figure 9, miR‐429 downregulated CRKL expression by selectively targeting *CRKL*‐3′‐UTR at the 3728‐3735 bp site, CRKL downregulation promoted the expression level of p‐Raf, p‐MEK and p‐ERK, then induces erythroid differentiation of K562 cells by increasing globin, haemoglobin and erythroid differentiation‐specific genes expression. Taken together, we have established a new functional role and molecular pathway for miR‐429‐CRKL axis during erythroid differentiation. These findings could be fundamental to the development of a novel potential diagnostic biomarker and therapeutic target for CML patients.

## 5 CONCLUSIONS

CRKL acts as tumour promotor and miR‐429 acts as a tumour suppressor in CML, their dysexpressions are involved in the carcinogenesis and progression of CML. We have uncovered miR‐429‐CRKL axis contributes to erythropoiesis of CML. The identified miR‐429‐CRKL axis provides new insight into the pathogenesis of CML and represents a potential therapeutic target for diagnosis and treatment of CML. Moreover, the different features of CRKII and CRKL indicate that they may serve differently in leukemogenesis. Our findings point to CRKL rather than CRKII as a biomarker associating with erythropoiesis of CML. Their different functions in CML cells may result from different preferential interactions with binding partners, thereby activating different signalling pathways leading to different roles in CML.

## AUTHOR CONTRIBUTIONS

**Chunmei Guo:** Funding acquisition (equal); investigation (equal); methodology (equal); writing – original draft (equal); writing – review and editing (equal). **Xinxin Lv:** Data curation (equal); investigation (equal); methodology (equal); validation (equal). **Qiuling Zhang:** Data curation (equal); investigation (equal); methodology (equal); validation (equal). **Lina Yi:** Investigation (equal). **Yingying Ren:** Investigation (equal). **Zhaopeng Li:** Investigation (equal). **Jinsong Yan:** Resources (equal). **Shanliang Zheng:** Investigation (equal). **Ming‐Zhong Sun:** Conceptualization (equal); resources (equal); supervision (equal); writing – review and editing (equal). **Shuqing Liu:** Conceptualization (equal); resources (equal); writing – review and editing (equal).

## CONFLICT OF INTEREST STATEMENT

The authors declare no conflicts of interest.

## Supporting information


Tables S1–S3

## Data Availability

The data support the findings of this study are available from the corresponding author upon reasonable request.
