# Supplementary material for: CRKL but not CRKII contributes to hemin‐induced erythroid differentiation of CML
Source: J Cell Mol Med. 2024 Apr 29;28(9):e18308. doi: 10.1111/jcmm.18308 (PMC11057422; doi:10.1111/jcmm.18308)
Supplement: Supplementary file 1 — Tables S1–S3 [file JCMM-28-e18308-s001.docx]

**Supplementary material:**

Table 1 Synthesized sequences of primers for targeting genes

| **Gene** |  | **Primer Sequence** |
| --- | --- | --- |
| *CRKL* | Forward | 5’-GTGCTTATGACAAGACTGCCT-3’ |
|  | Reverse | 5’-CACTCGTTTTCATCTGGGTTT-3’ |
| *CRKII* | Forward | 5’-CTATGCCCAACCCAGCGTCA-3’ |
|  | Reverse | 5’-CGTTTGCCATTACACTCCCC -3’ |
| *α-globin* | Forward | 5’-GTCAACTTCAAGCTCCTAAGC-3’ |
|  | Reverse | 5’-TGGACAAGTTCCTGGCTTCTG-3’ |
| *γ-globin* | Forward | 5’-GCAGCTTGTCACAGTGCAGTTC-3’ |
|  | Reverse | 5’-TGGCAAGAAGGTGCTGACTTC-3’ |
| *ε-globin* | Forward | 5’-CCAGACAGAGAGGCAGCAGC-3’ |
|  | Reverse | 5’-TCCAGGGGTAAACAACGAGG-3’ |
| *GATA-1* | Forward | 5’-CTGCGGCCTCTATCACAAGATG-3’ |
|  | Reverse | 5’-ACTGAGTACCTGCCCGTTTACTGAC-3’ |
| *HMGB2* | Forward | 5’- TGTCCTCGTACGCCTTCTTC-3’ |
|  | Reverse | 5’-CCTCCTCATCTTCTGGTTCG-3’ |
| *GPA* | Forward | 5'-GACAAATGATACGCACAAACGG-3' |
|  | Reverse | 5'-TCCAATAACACCAGCCATCAC-3' |
| *HBA* | Forward | 5'-TGGAGGGTGGAGACGTCCTG-3' |
|  | Reverse | 5'-TCCATCCCCTCCTCCCGCCCCTGCCTTTTC-3' |
| *ACTB* | Forward | 5’-AGGCCAACCGCGAGAAG-3’ |
|  | Reverse | 5’-ACAGCCTGGATAGCAACGTACA-3’ |
| *GAPDH* | Forward | 5'-GGT GAA GGT CGG TGT GAA CG-3' |
|  | Reverse | 5'-AAGTGGTCGTTGAGGGCAATG-3' |

Table 2 Gene microarray screened differentially expressed genes

in K562-shRNA-CRKL and K562-shRNA-NC cells

| **Probe Set ID** | **Gene Symbol** | **Description** | **Fold change*** | **Style** |
| --- | --- | --- | --- | --- |
| TC11001331.hg.1 | *HBD* | Hemoglobin, delta | 1.6 | up |
| TC16000008.hg.1 | *HBA1* | Hemoglobin, alpha1 | 2.2 | up |
| TC16000007.hg.1 | *HBA2* | Hemoglobin, alpha2 | 2.3 | up |
| TC16002032.hg.1 | *HBZ* | Hemoglobin, zeta | 2.5 | up |
| TC01003378.hg.1 | *SPTA1* | Spectrin, alpha, erythrocytic 1 | 1.5 | up |
| TC14001220.hg.1 | *SPTB* | Spectrin, beta, erythrocytic | 1.6 | up |
| TC03002155.hg.1 | *TFRC* | Transferring receptor (p90, CD71) | 1.8 | up |
| TC01003312.hg.1 | *PKLR* | Pyruvate kinase, liver and RBC | 1.8 | up |
| TC18000300.hg.1 | *EPB41L3* | Erythrocyte membrane protein band 4.1-like 3 | 1.9 | up |
| TC16000380.hg.1 | *AHSP* | Alpha hemoglobin stabilizing protein | 2.2 | up |
| TC01000318.hg.1 | *RHD* | Rh blood group, D antigen | 1.9 | up |
| TC01002377.hg.1 | *RHCE* | Rh blood group, CcEe antigens | 2.1 | up |

*Refers to mRNA level changes of deregulated genes in K562-shRNA-CRKL cells compared with K562-shRNA-NC cells.

Table 3 iTRAQ quantitative proteomic screened differentially expressed proteins

in K562-shRNA-CRKL and K562-shRNA-NC cells

| **Accession** | **Protein Symbol** | **Description** | **Fold change*** | ***P*** |
| --- | --- | --- | --- | --- |
| P46109 | CRKL | v-crk sarcoma virus CT10 oncogene homolog (avian)-like | 0.6 | 0.0092 |
| P02100 | HBE1 | Hemoglobin, epsilon1 | 1.2 | 0.0071 |
| P02042 | HBD | Hemoglobin, delta | 1.3 | 0.0061 |
| P02008 | HBZ | Hemoglobin, zeta | 1.2 | 0.0034 |
| D9YZU8 | HBG1 | Hemoglobin, gamma A | 1.2 | 0.0080 |
| B7WNQ9 | GATA-1 | Erythroid transcription factor | 1.3 | 0.0326 |
| P26583 | HMGB2 | High mobility group protein B2 | 1.2 | 0.0311 |
| P30613 | PKLR | Pyruvate kinase, liver and RBC | 1.2 | 0.0086 |

*Refers to protein level changes of deregulated genes in K562-shRNA-CRKL cells compared with K562-shRNA-NC cells.
